# Safety of cangrelor and transition to oral P2Y_12_ inhibitors in patients undergoing percutaneous coronary intervention: the ARCANGELO study

**DOI:** 10.1093/ehjopen/oead076

**Published:** 2023-08-28

**Authors:** Leonardo De Luca, Paolo Calabrò, Piera Capranzano, Carlo Di Mario, Fabio Chirillo, Cristina Rolfo, Alberto Menozzi, Maurizio Menichelli, Leonardo Bolognese, Giuseppe Musumeci

**Affiliations:** Division of Cardiology, Department of Cardiosciences, Azienda Ospedaliera San Camillo-Forlanini, Circonvallazione Gianicolense, 87, 00152 Roma, Italy; UOC Cardiologia Clinica con UTIC. A.O.R.N. Sant'Anna e San Sebastiano, Caserta, Italy; Cardiology Division, Policlinico Hospital, University of Catania, Catania, Italy; Interventistica Cardiologica Strutturale A.O.U. Careggi, Firenze, Italy; UOC Cardiologia, Ospedale San Bassiano, Bassano del Grappa (VI), Italy; Cardiologia Ospedale degli Infermi, Rivoli, Italy; S.C. Cardiologia Ospedale S. Andrea, La Spezia, Italy; Cardiologia Ospedale Fabrizio Spaziani, Frosinone, Italy; Cardiologia Ospedale San Donato, Arezzo, Italy; SC Cardiologia, AO Ordine Mauriziano, Torino, Italy

**Keywords:** Cangrelor, Acute coronary syndrome, Bleeding, P2Y_12_ receptor inhibitor, Percutaneous coronary intervention

## Abstract

**Aims:**

Cangrelor is the only intravenous P2Y_12_ inhibitor available. Safety, efficacy, and transitioning from cangrelor to oral P2Y_12_ inhibitors were recorded in patients with acute coronary syndrome (ACS). The ARCANGELO study aims to assess the safety of cangrelor on bleeding and the effects of the transition to oral P2Y_12_ inhibitors in a real-world setting according to the European Medical Agency’s requirement.

**Methods and results:**

Adult patients with ACS undergoing percutaneous coronary intervention (PCI) receiving cangrelor were included in the study. Patients were followed for 30 days. Incidence of bleeding events, major adverse cardiac events, and transition strategy to oral P2Y_12_ were recorded. Among 1004 ACS patients undergoing PCI, 995 (99.1%) were eligible for the analysis; 597 (60.0%) of them had ST-segment elevation myocardial infarction. A total of 925 (93.1%) patients underwent PCI by radial catheter access, and 972 (97.2%) received drug-eluting stents. All eligible patients received bolus and cangrelor infusion between 2 and 4 h in 95% of the cases. A total of 730 patients (73.4%) received ticagrelor, 127 (12.8%) prasugrel, and 138 (13.9%) clopidogrel as transition therapy. Bleeding, according to Bleeding Academic Research Consortium (BARC) criteria, within 30 days post-PCI occurred in 5.2% of patients (95% confidence interval: 3.9–6.8%); 0.5% experienced a moderate (BARC 3), and all others mild (BARC 1–2) bleeding events. Major adverse cardiac events occurred in 14 (1.4%) patients, principally all-cause mortality (*n* = 6 patients) and myocardial infarction (*n* = 7 patients).

**Conclusion:**

The use of cangrelor in ACS patients undergoing PCI and the transition strategy to P2Y_12_ inhibitors are confirmed as safe and effective in daily practice.

## Graphical Abstract

**Graphical Abstract:**
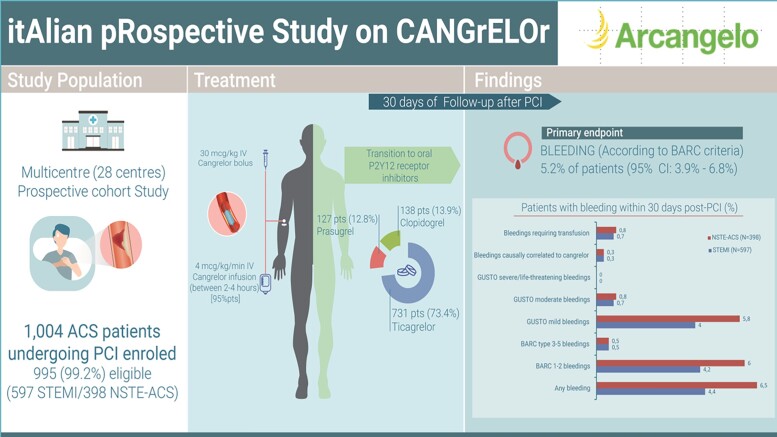
ACS, acute coronary syndrome; BARC, bleeding academic research consortium; GUSTO, global use of strategies to open occluded coronary arteries; NSTE-ACS, non-ST-elevation acute coronary syndromes; PCI, percutaneous coronary intervention; STEMI: ST-segment elevation myocardial infarction.

## Introduction

The use of oral platelet P2Y_12_ receptor inhibitors in association with acetylsalicylic acid [dual antiplatelet therapy (DAPT)] has consistently been shown to reduce the risk of thrombotic complications related to percutaneous coronary intervention (PCI)^1^ and is considered standard therapy in patients with coronary artery disease (CAD).^2,3^ Even if DAPT is considered the standard of therapy, monotherapy with P2Y_12_ receptor inhibitors could be useful in complex procedures.^4^ The delayed onset of action of oral P2Y_12_ inhibitors and lack of bioavailability in critical patients limit their use in case of urgent PCI.^5^ Furthermore, nausea has been reported in almost two-thirds and vomiting in nearly one-third of patients with ST-segment elevation myocardial infarction (STEMI)^1^ impairing oral P2Y_12_ administration.^6,7^ These limitations may be a major concern in patients with acute coronary syndrome (ACS) undergoing emergency PCI due to the high risk of procedural thrombotic complications.^8^ Cangrelor is the only intravenous P2Y_12_ receptor inhibitor available for the reduction of thrombotic cardiovascular events in adult patients with CAD undergoing PCI who have not received an oral P2Y_12_ inhibitor before the PCI procedure and in whom oral periprocedural therapy with P2Y_12_ inhibitors is not feasible or desirable.^9^ Its quick onset and offset of action, short half-life (3–5 min), and reversible binding properties allow for overcoming many limitations of the oral platelet P2Y_12_ receptor inhibitors.^8,10–12^ The benefit of cangrelor over clopidogrel has been demonstrated in the CHAMPION programme.^13–18^ During the clinical development programme of cangrelor, ticagrelor, and prasugrel were not marketed, and, therefore, the CHAMPION studies were designed and conducted only in patients transitioning from cangrelor to clopidogrel.

Several pharmacokinetic and pharmacodynamic studies, as part of the cangrelor clinical development programme, have shown that the inhibition of platelet aggregation is maintained when a transition from cangrelor to any P2Y_12_ inhibitors, including clopidogrel, ticagrelor, and prasugrel, is carried out.^19–22^ Moreover, the efficacy and the safety of a transition from cangrelor to ticagrelor or prasugrel have been confirmed in several studies published over the years,^23–25^ and the use of cangrelor has been endorsed by the latest European Society of Cardiology guidelines.^26^

Nonetheless, given that ticagrelor and prasugrel are the preferred P2Y_12_ inhibitors in the treatment of ACS patients in current clinical practice, the collection of additional information in the itAlian pRospective Study on CANGrELOr (ARCANGELO) was aimed at prospectively assessing the safety profile of cangrelor and the management of transitions to oral P2Y_12_ inhibitors in real-world clinical practice.

The aim of the multicentre, observational, prospective cohort study ARCANGELO study was the evaluation of the safety and effectiveness of cangrelor when administered to patients with ACS undergoing PCI in a real-world setting, including patients who received cangrelor transitioning to either clopidogrel, prasugrel, or ticagrelor.

## Methods

The ARCANGELO observational study is a non-imposed post-authorization safety study (PASS) category 3,^27^ registered before the beginning of the patient’s enrolment in October 2020 (registration number NCT04471870)^28^ as part of the cangrelor risk management plan. The study protocol and preliminary results from the ARCANGELO study’s interim analysis were presented in a previous article,^28^ showing a good safety profile.

The design of this cohort study required the inclusion of a total of 1000 consecutive adults (age ≥ 18 years), eligible to PCI for ACS who received cangrelor and who consented to take part in the study in 28 Italian centres.^26,27^

The definitions of ACS and their differentiation into ST-segment-elevation myocardial infarction (STEMI), non-ST-elevation myocardial infarction (NSTEMI), and unstable angina followed the standards proposed by the fourth universal definition of myocardial infarction (MI).^29^

The primary outcome of the study was the incidence of any bleedings, defined according to Bleeding Academic Research Consortium (BARC) criteria and calculated as the ratio between the number of patients experiencing at least one event during the 30-day observation period over the total number of examined patients.^30^

The secondary outcomes included the incidence of bleedings distinguished according to their severity based on the BARC and the Global Use of Strategies to Open Occluded Coronary Arteries (GUSTO) criteria.^31^ The incidence of major adverse cardiac events (MACEs), a composite endpoint including death, MI, ischaemia-driven revascularization (IDR), and stent thrombosis (ST), was also a secondary endpoint, collected post-PCI. The proportion of patients receiving any of the oral platelet P2Y_12_ receptor antagonists was evaluated as well as the type and timing of administration of any GPI IIb/IIIa inhibitors during observation.

Moreover, treatment-emergent adverse events (TEAEs) were assessed; net adverse clinical events (NACEs; including BARC 3–5 bleedings or MACE events) were analysed post hoc.

All the analyses were stratified separately by STEMI vs. non–ST-elevation acute coronary syndrome (NSTE-ACS) final diagnosis.

### Statistical analysis

The results of the statistical analyses were summarized by descriptive statistics including frequency count and percentage for categorical variables and the number of observations, mean ± standard deviation (SD), median, and interquartile range (IQR), when appropriate, for continuous variables.

Statistical testing for differences between STEMI and NSTE-ACS groups was performed on all the eligible patients and was exploratory. It was performed only when stratified results were relevantly different, or when the tested variable was deemed to be relevant for the interpretation of results. Statistical tests were performed using the Wilcoxon test for continuous variables and the *χ*^2^ or Fisher exact test, as appropriate, for categorical variables, using the SAS software. Missing data were not replaced or included in the analyses.

## Results

**Figure 1:**
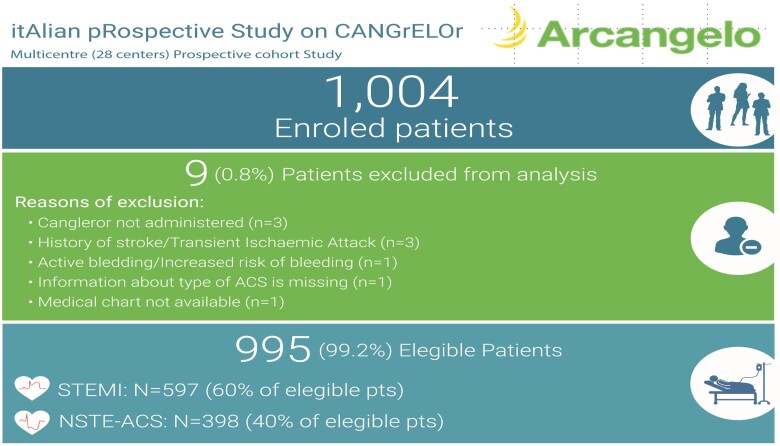
Patient disposition of the ARCANGELO study. NSTE-ACS, non–ST-segment elevation acute coronary syndromes; STEMI, ST-segment elevation myocardial infarction.

A total of 1004 patients were enroled from October 2020 to January 2022, in 28 Italian centres. Among the enroled subjects, all but nine patients met the selection criteria; therefore, 995 (99.1%) patients received cangrelor and were eligible for the analyses (*Figure 1*). Overall, 967 patients (97.2% of eligible patients) completed the study, because 28 (2.8%) discontinued prematurely: 22 subjects were lost to follow-up, and 6 died before completing the 30-day prospective observational period. At the time of cangrelor administration, 597 (60.0%) of the eligible patients were diagnosed with STEMI and 398 (40.0%) with NSTE-ACS, 272 (27.3%) with NSTEMI and 126 (12.7%) with unstable angina (*Figure 1*). The median (IQR) duration of observation from cangrelor administration to the final study visit was 31 days (30–32 days). The median age was 65 years (IQR 56–73), and 21.6% of the patients (*n* = 215) were ≥75 years old.

The majority (83.2%) of eligible patients had at least one comorbidity; the most frequent was hypertension (66.0%), followed by dyslipidaemia (47.1%) and diabetes mellitus (20.7%). Among other relevant comorbidities, 4.4% of the patients (*n* = 44) had chronic obstructive pulmonary disease, 4.5% (*n* = 45) gastrointestinal disorder peripheral arterial occlusive disease (*n* = 38, 3.8%), and 3.2% (*n* = 32) renal/urinary disorders (mainly chronic kidney disease *n* = 23, 2.3%) (*Table 1*).

**Table 1:** Demographic and clinical characteristics of the eligible patients

		STEMI	NSTE-ACS	Total
		(*n* = 597)	(*n* = 398)	*n* = 995
Age at enrolment (years)*	Mean (SD)	63.8 (11.6)	66.0 (11.4)	64.7 (11.5)
	Median (IQR)	63.0 (56–72)	66.0 (57–75)	65.0 (56–73)
Age at enrolment (classes) [*n* (%)]**	<75 years	484 (81.1%)	296 (74.4%)	780 (78.4%)
	≥75 years	113 (18.9%)	102 (25.6%)	215 (21.6%)
Gender [*n* (%)]	Male	469 (78.6%)	302 (75.9%)	771 (77.5%)
	Female	128 (21.4%)	96 (24.1%)	224 (22.5%)
Weight (kg)	Mean(SD)	80.7 (15.2)	79.4 (14.2)	80.2 (14.8)
	Median (IQR)	80 (70–89)	79 (70–87)	80 (70–88)
Obese [BMI (kg/cm^2^) ≥ 30]		126 (21.1%)	95 (23.9%)	221 (22.2%)
Smoking habits (current smokers)		240 (42.0%)	122 (31.2%)	362 (37.6%)^a^
Family history of CAD		157 (31.2%)	121 (37.1%)	278 (33.5%)^b^
Prior MI		42 (7.0%)	45 (11.3%)	87 (8.7%)
Prior neoplasms (any)		15 (2.5%)	22 (5.5%)	37 (3.7%)
Prior interventional procedure in the previous year [*n* (%)]***		13 (2.2%)	21 (5.3%)	34 (3.4%)
Risk factors and comorbidities [*n* (%)]^c^	Any	466 (78.1%)	362 (91.0%)	828 (83.2%)
	Hypertension	360 (60.3%)	297 (74.6%)	657 (66.0%)
	Hyperlipidaemia	224 (37.5%)	245 (61.6%)	469 (47.1%)
	Diabetes	113 (18.9%)	93 (23.4%)	206 (20.7%)
	Gastrointestinal disorders (any)	20 (3.4%)	25 (6.3%)	45 (4.5%)
	COPD	18 (3.0%)	26 (6.5%)	44 (4.4%)
	Peripheral artery disease	14 (2.3%)	24 (6.0%)	38 (3.8%)
	Atrial fibrillation	18 (3.0%)	18 (4.5%)	36 (3.6%)
	Chronic kidney disease	14 (2.3%)	9 (2.3%)	23 (2.3%)

Comparisons of STEMI vs. NSTE-ACS were performed with Wilcoxon, *χ*^2^, or Fisher’s exact test, as appropriate.

COPD, chronic obstructive pulmonary disease; IQR, interquartile range; *N*, number of patients; NSTE-ACS, non–ST-elevation acute coronary syndromes; SD, standard deviation; STEMI, ST-segment elevation myocardial infarction; MI, myocardial infarction.

a The percentage was calculated on a sample of 963 patients.

b The percentage was calculated on a sample of 830 patients.

c The same patient could have more than 1 comorbidity; *P = 0.0028; **P = 0.0119; ***P = 0.0117.

Roughly half of the patients (*n* = 509, 51.2%) had a multi-vessel coronary disease [296 (49.6%) STEMI; 213 (53.5%) NSTE_ACS], involving two vessels in 59.3% (*n* = 302) of the cases. In terms of the location of the culprit coronary occlusion, the left anterior descending coronary artery was the most frequently involved vessel, accounting for 70.1% of the subjects (*n* = 697). A radial artery access was used in most of the patients (*n* = 926, 93.1%), and drug-eluting stents (DES) were implanted in almost all the patients (*n* = 972, 97.7%).

All patients included in the analysis were treated with cangrelor, receiving a 30 µg/kg intravenous bolus immediately followed by a 4 µg/kg/min intravenous infusion (as per the summary of product characteristics [SmPC]).

According to investigators’ indication, cangrelor was chosen because of the urgency of PCI in 888 (89.2%) patients, the presence of nausea/vomiting (42–4.2%), or difficulties in swallowing (25–2.5%) (the complete list is available in Supplementary material online, *Table S1*).

Nearly all patients received aspirin 24 h before or during PCI (991–99.6%), while 877 (88.1%) were treated with heparin. The median (IQR) duration of cangrelor infusion was 122.0 (120–164) min, with no significant difference when comparing patients with STEMI (122 [120–160] min) or NSTE-ACS (120.5 [120–170] min) (*P* = 0.4318); infusions lasted 2–4 h for 95.0% of the subjects (*n* = 933) (*Table 2*).

**Table 2:** Details of acute coronary syndrome and percutaneous coronary intervention and use of cangrelor

		STEMI	NSTE-ACS	Evaluable patients
		(*n* = 597)	(*n* = 398)	*n* = 995
Number of coronary vessels^	Mono-vessel	301 (50.4%)	185 (46.5%)	486 (48.8%)
	Multi-vessel	296 (49.6%)	213 (53.5%)	509 (51.2%)
	2 vessels	177 (59.8%)	125 (58.7%)	302 (59.3%)
	3 vessels	99 (33.4%)	76 (35.7%)	175 (34.4%)
	≥4 vessels	20 (6.8%)	12 (5.6%)	32 (6.3%)
Coronary vessel [*n* (%)]^a^	LAD coronary artery	423 (70.9%)	274 (68.8%)	697 (70.1%)
	Left circumflex artery	225 (37.7%)	190 (47.7%)	415 (41.7%)
	Right coronary artery	326 (54.6%)	195 (49.0%)	521 (52.4%)
Main characteristics of the index PCI				
Duration of PCI (min)	Mean (SD)	46.9 (37.0)	50.0 (34.3)	48.1 (36.0)
	Median (IQR)	40.0 (25.0–60.0)	45.5 (27.0–70.0)	40.0 (25.0—63.0)
Catheter access site(s) [*n* (%)]^a^	Radial	552 (92.5%)	374 (94.0%)	926 (93.1%)
	Femoral	50 (8.4%)	28 (7.0%)	78 (7.8%)
	Brachial	50 (8.4%)	28 (7.0%)	78 (7.8%)
	Ulnar	0 (0.0%)	1 (0.3%)	1 (0.1%)
Patient distribution by type of stenting implantation [*n* (%)]^a^	No stent implantation	10 (1.7%)	9 (2.3%)	19 (1.9%)
	Any	587 (98.3%)	389 (97.7%)	976 (98.1%)
	−DES	584 (97.8%)	388 (97.5%)	972 (97.7%)
	None	10 (1.7%)	9 (2.3%)	19 (1.9%)
Patient distribution by number of vessels with at least one DES implanted [*n* (%)]	1 vessel	499 (83.6%)	300 (75.4%)	799 (80.3%)
	2 vessels	72 (12.1%)	74 (18.6%)	146 (14.7%)
Patients with planned staged PCIs [*n* (%)]	Yes	153 (25.8%)	59 (14.9%)	212 (21.3%)
Use of cangrelor and peri-procedural antithrombotic treatments				
Duration of treatment (min)	Mean (SD)	152.4 (136.1)	143.9 (36.3)	148.8 (106.2)
	Median (IQR)	122.0 (120.0–160.0)	120.5 (120.0–170.0)	122.0 (120–164)
Duration of treatment by classes (h)	<2 h	17 (2.8%)	18 (4.5%)	35 (3.5%)
	2–4 h	557 (93.3%)	376 (94.5%)	933 (93.8%)
	>4 h*	10 (1.7%)	4 (1.0%)	14 (1.4%)
	UNK	13 (2.2%)	0 (0.0%)	13 (1.3%)
Most frequent antithrombotic treatments [*n* (%)]*	Acetylsalicylic acid	594 (99.5%)	397 (99.7%)	991 (99.6%)
	GPI (IIB/IIIA)	25 (4.2%)	1 (0.3%)	26 (2.6%)
	Heparin	515 (86.3%)	362 (91.0%)	877 (88.1%)
	Fondaparinux	11 (1.8%)	48 (12.1%)	59 (5.9%)
	Direct factor Xa inhibitors	7 (1.2%)	11 (2.8%)	18 (1.8%)
	Direct thrombin inhibitors	(0.0%)	4 (1.0%)	4 (0.4%)

CAD, coronary artery disease; COPD, chronic obstructive pulmonary disease; DES, drug-eluting stent; GPI IIB/IIIA, glycoprotein IIb/IIIa inhibitor; IQR, interquartile range; LAD, left anterior descending; *n*, number of patients; NSTE-ACS, non–ST-elevation acute coronary syndromes; PCI, percutaneous coronary intervention; SD, standard deviation; STEMI, ST-segment elevation myocardial infarction.

a The same patient could have more than 1 option; *P ≤ 0.0001; ^*P* = 0.2236.

In 26 (2.6%) patients, a GPI IIb/IIIa was administered in the peri-procedural setting, primarily in STEMI patients (*n* = 25) (*Table 2*). Slow flow/no-reflow (*n* = 12, 1.2%), large thrombus (*n* = 10, 1.0%), and other thrombotic complications (*n* = 4, 0.4%) were the main reasons for their use.

**Figure 2:**
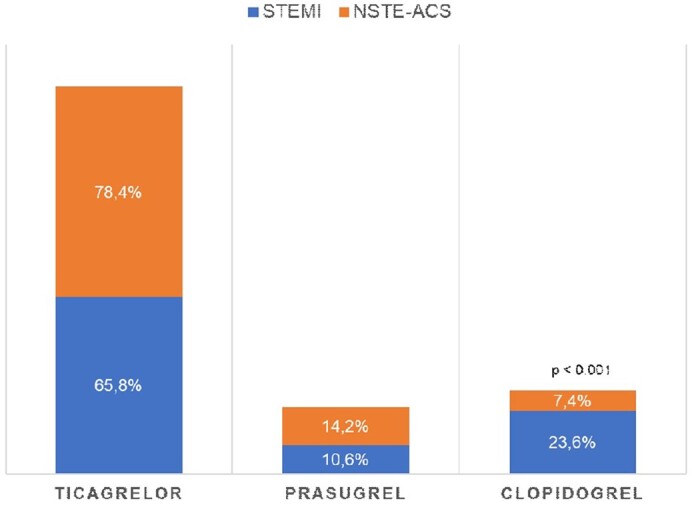
Transition from cangrelor to oral platelet P2Y_12_ inhibitor. NSTE-ACS, non–ST-elevation acute coronary syndromes; STEMI, ST-segment elevation myocardial infarction.

All patients received at least one oral platelet P2Y_12_ receptor antagonist as a transition strategy from cangrelor: 730 (73.4%) received ticagrelor, 138 (13.9%) received clopidogrel, and 127 (12.8%) received prasugrel. Ticagrelor and prasugrel were used in similar proportions in STEMI and NSTE-ACS patients, while clopidogrel was mainly used in NSTE-ACS patients (*Figure 2*). Most of the transitions from cangrelor to a P2Y_12_ receptor inhibitor were performed in compliance with the recommendations included in cangrelor EU SmPC as summarized in descriptive statistics (*Table 3*).

**Table 3:** Timing for the transition from cangrelor to oral platelet P2Y_12_ inhibitor

		STEMI	NSTE-ACS	Total evaluable
		*n*	*n*	*n*
		Median (IQR)	Median (IQR)	Median (IQR)
Time to transition from the start of cangrelor (min)	Clopidogrel	*n* = 39	*n* = 88	*n* = 127
		125 (120–240)	130 (120–180)	130 (120–180)
	Prasugrel	*n* = 79	*n* = 42	*n* = 121
		120 (100–143)	120 (120–140)	120 (108–140)
	Ticagrelor	*n* = 386	*n* = 245	*n* = 631
		120 (92–140)	120 (100–151)	120 (95–150)
Time to transition from the end of cangrelor (min), when the transition was done before cangrelor end	Clopidogrel	*n* = 1	*n* = 1	*n* = 2
		20 (20–20)	30 (30–30)	25 (20–30)
	Prasugrel	*n* = 35	*n* = 10	*n* = 45
		30 (30–30)	30 (15–30)	30 (30–30)
	Ticagrelor	*n* = 193	*n* = 97	*n* = 290
		30 (30–87)	30 (30–30)	30 (30–75)
Time to transition from the end of cangrelor (min), when the transition was done after cangrelor end	Clopidogrel	*n* = 39	*n* = 88	*n* = 127
		0 (0–30)	0 (0–0)	0 (0–5)
	Prasugrel	*n* = 44	*n* = 32	*n* = 76
		0 (0–0)	0 (0–1.5)	0 (0–0)
	Ticagrelor	*n* = 212	*n* = 148	*n* = 360
		0 (0–5)	0 (0–0)	0 (0–1)

Sixteen patients received ticagrelor before the initiation of the cangrelor infusion.

### Bleeding events

**Figure 3:**
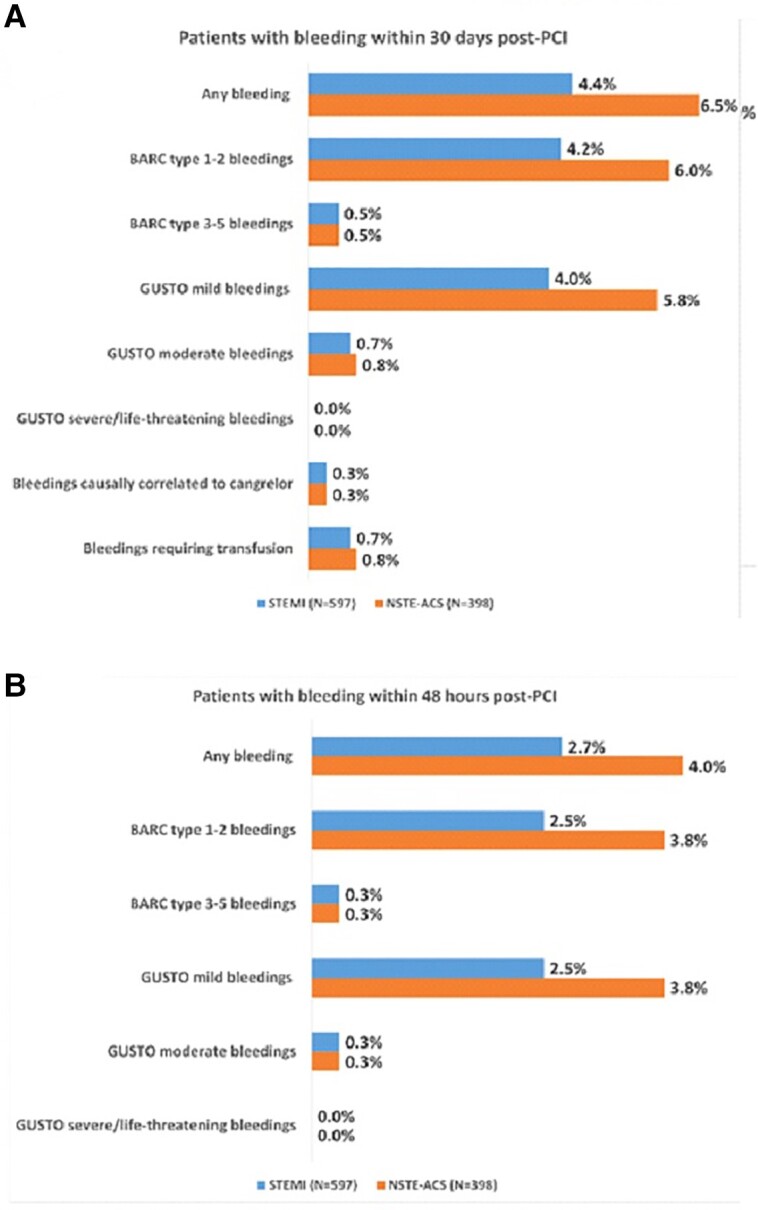
Incidence of bleeding events (% of patients) at 48 h (*A*) and 30 days (*B*) by Bleeding Academic Research Consortium and Global Use of Strategies to Open Occluded Coronary Arteries classifications. BARC, Bleeding Academic Research Consortium; GUSTO, Global Use of Strategies to Open Occluded Coronary Arteries; NSTE-ACS, non–ST-elevation acute coronary syndromes; PCI, percutaneous coronary intervention; STEMI, ST-segment elevation myocardial infarction; *N*, number of patients.

Fifty-two patients (5.2%; 95% confidence interval [CI: 3.9–6.8%]) experienced at least one bleeding event during the 30 days of observation, for a total of 55 bleeding events (incidence density rate = 5.48 events over 100 person-months). Bleedings occurred in 26 STEMI patients (4.4%; 95% CI: 2.9–6.3%) and 26 NSTE-ACS patients (6.5%; 95% CI: 4.3–9.4%) (*P* = 0.15) (*Figure 3A*). In the 48 (±24) h from PCI, 32 (3.2%) patients experienced bleeding (16 [2.7%] with STEMI and 16 [4.0%] with NSTE-ACS; *Figure 3*), and BARC type 3–5 (moderate–severe) bleedings were experienced by five patients (0.5%), all BARC type 3 and none a type 4 or 5 event; 3 of whom (0.3%) had events within 48 h post-PCI. The BARC type 1–2 (mild) bleedings were experienced in 49 (4.9%; 25 [4.2%] STEMI and 24 [6.0%] NSTE-ACS) patients during the whole observation period; 30 (3.0%) of them had events within 48 h post-PCI. Most of these bleeding events (*n* = 37, 74.0%) were classified as BARC type 1; 24 (2.4%) of them occurred within 48 h post-PCI. The most frequent bleeding was mild ecchymosis (*n* = 12–24%); details on bleeding location observed during the study, by severity, are available in Supplementary material online, *Table S2*.

Bleedings occurred in 3 (2.2%) of the patients who were using clopidogrel, in 10 (7.9%) of those treated with prasugrel, and in 39 (5.3%) of those treated with ticagrelor (see Supplementary material online, *Table S3*).

During the study, mild bleedings, according to GUSTO criteria, were observed in 47 (4.7%) patients [24 (4.0%) STEMI and 23 (5.8%) NSTE-AC, respectively] and for 30 (3.0%) of whom occurring within 48 h post-PCI. A total of 7 [0.7%; 4 STEMI (0.7%) and 3 (0.8%) NSTE-ACS] patients had moderate bleeding events requiring blood transfusion but not resulting in haemodynamic compromise by GUSTO criteria; 3 (0.3%) of them occurred within the 48 h post PCI.

During the entire observation period, bleeding events were principally correlated to PCI (as judged by investigators) in 34 (3.4%) patients or to other concomitant drugs in 28 (2.8%) patients, while bleeding causally related to cangrelor, along with other causes, occurred in 3 (0.3%) (2 [0.3%] STEMI and 1 [0.3%] NSTE-ACS) patients, for a total of 5 bleeding events.

### Major adverse cardiac events

A total of 14 out of 995 eligible patients (1.4%) had at least one MACE (including death, MI, IDR, and ST) within 30 days post-PCI (10 of 597 [1.7%] STEMI and 4 out of 398 [1.0%] NSTE-ACS); the majority of the patients (10 out of 14) experienced a MACE within the first 48 h. The calculated incidence density rate for any MACE accounted for 1.49 events over 100 person-months. MI, which occurred in 7 (0.7%) patients, and all-cause death, observed in 6 (0.6%), were the most frequent MACEs. Four of the fatal events occurred within 48 h post PCI. Two patients (0.2%) experienced at least one ST; moreover, one event of ST was fatal. There have been no reports of IDR (see Supplementary material online, *Table S4*).

### Incidence of net adverse clinical events

A NACE was experienced by 19 (1.9%) patients during the observation (13 [2.2%] STEMI and 6 [1.5%] NSTE-ACS). The mean (SD) time to the first NACE was 4.7 (6.6) days (5.7 [7.5] days for STEMI and 2.7 [3.4] days for NSTE-ACS).

At least one TEAE was experienced by 226 (22.7%) of the patients (160 [26.8%] STEMI and 66 [16.6%] NSTE-ACS). Of TEAEs, 72 were serious and occurred in 53 patients (5.3%). Cardiac disorders were the most frequently reported TEAEs, experienced by 88 (8.8%) patients. According to the investigators’ assessment, eight TEAEs were related to cangrelor and occurred in 6 (0.6%) patients; all of them did not reveal any new or significant concerns about cangrelor safety and were resolved by the end of the observation period (see Supplementary material online, *Table S5*).

## Discussion

The ARCANGELO study showed that using cangrelor in patients with ACS undergoing PCI and then transitioning to an oral P2Y_12_ inhibitor is a safe and effective practice. The rate of bleeding, thrombotic complications, and adverse drug reactions was low. The incidence of patients who experienced bleeding was 3.2% of the eligible patients within 48 h post-PCI and 5.2% over the whole 30-day observation period according to BARC criteria. The evaluation of the bleeding events with the GUSTO scale provided very similar results. Notably, according to CHAMPION pooled analysis,^32^ bleeding events at 48 h post-PCI (GUSTO criteria) were reported in 17.5% of the patients treated with cangrelor, of whom 0.2% experienced severe/life-threatening bleedings, 0.8% severe/moderate, and 0.6% moderate, whereas none of the patients from the ARCANGELO study had any severe events, and 0.7% had a moderate bleeding event. Furthermore, a lower incidence of mild bleeding events (4.7%) was observed in the ARCANGELO study than in the CHAMPION population where mild bleeding occurred in 16.8% of patients.^32^ In FABOLUS-FASTER trial comparing the tirofiban and cangrelor with chewed or integral prasugrel in STEMI patients undergoing PCI, BARC moderate or severe bleeding has been observed in 10% of the patients treated with cangrelor, in 5% of those treated with tirofiban, and in 10.1% of those treated with chewed prasugrel in the 30 days.^33^ The low rates of bleeding observed in the ARCANGELO study also confirm the findings from other real-world studies. One study performed at a tertiary care hospital revealed a total of 18 mild-to-moderate bleeding events in the 147 examined patients, whereas severe, life-threatening, or intracranial bleeding was not observed.^34^ These findings could be influenced by the overall improvement in PCI management over the years such as the vascular access site selection. Thus, the use of the radial artery in 93.1% of ARCANGELO patients reflects the implementation of current coronary artery revascularization guidelines^35^ vs. PCI management during the CHAMPION programme.^32^ In terms of effectiveness, the rate of patients who experienced MACEs was 1.4% in the 30 days post-PCI in the ARCANGELO study. A comprehensive meta-analysis,^36^ the results of the CHAMPION PHOENIX trial,^37^ and the pooled CHAMPION clinical trials^32^ consistently report higher rates of MACEs after cangrelor use. One possible explanation is the overall improved performance of current PCI and stenting equipment and operator expertise gained over 10 years of systematic PCI in ACS.^38,39^ In the ARCANGELO study, DES were implanted in 97.7% of the patients. In contrast, only half of the patients in the CHAMPION PHOENIX trial (55.6%) and from the CHAMPION trials’ pooled patients (53.2%) had DES implanted.^32,37^

The most recent analysis on the use of antiplatelet therapy in Italian coronary care units was performed in March 2014^40^ when cangrelor was not yet available. The results of the ARCANGELO study confirm the change in the PCI procedures in daily clinical practice in Italy, with GPIs used in only 2.6% and direct thrombin inhibitors in 0.4% of the patients. Similarly, the concomitant administration of aspirin (99.6% of the eligible patients) and heparins (88.1%) observed in the ARCANGELO study is consistent with established guidelines with the goal of reducing thrombotic complications during the peri-procedural period,^35^ which seems to contribute to a more standardized and clinically effective approach.

The results of the ARCANGELO study support the findings of other observational studies that show cangrelor is more commonly used in STEMI patients.^11,23–25,41^ A recent publication reported the interim findings from the US CAMEO registry, a retrospective observational study of platelet inhibition strategies for patients with MI undergoing PCI, which looked at the transition strategy from cangrelor to an oral P2Y_12_ inhibitor in real-world practice.^25^ The CAMEO study’s findings appear to be consistent with those of the ARCANGELO study about the use of ticagrelor, being the most frequently used P2Y_12_ inhibitor for a transition. The results from pharmacodynamic analyses of the POMPEII Registry suggested a lower platelet inhibition after cangrelor infusion cessation and transition to clopidogrel or prasugrel as compared with ticagrelor in complex PCI.^42^

The incidence of bleeding events within the first 48 h of MI hospitalization was 4.5%, and the incidence of MACE within the first 48 h was 6.3%, both lower when compared to patients who were not transitioned to an oral P2Y_12_ inhibitor.^25^

The main limitations of the ARCANGELO include that it was an observational study in which participants were enroled in 28 high-volume Italian centres with a 24-h available catheter laboratory; therefore, they were not randomly chosen among the entire pool of Italian sites. This is a single-arm study of patients treated with cangrelor, without a control group, as required by the regulatory authority (non-imposed category 3 safety study). Furthermore, an independent medical review of patients’ clinical data was not performed; however, the operational definitions of bleeding severity were deemed to be objective evaluations that do not require an independent medical review.

## Conclusions

The ARCANGELO study demonstrates that real-world use of cangrelor is mainly in patients with STEMI patients with ACS undergoing PCI, and then transitioning to an oral P2Y_12_ inhibitor is safe and effective. No significant or new concerns regarding the safety profile of cangrelor emerge from the ARCANGELO regardless of the oral P2Y_12_ inhibitor chosen for the transition strategy in the current era of coronary revascularization.

## Lead author biography


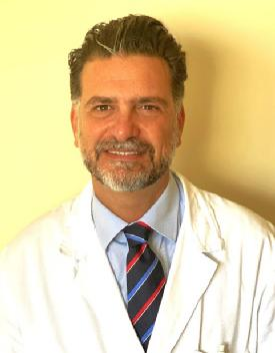
Leonardo De Luca, MD, PhD, FESC, FACC, is a critical care and interventional cardiologist at Azienda Ospedaliera San Camillo-Forlanini in Rome, Italy. He received his MD and PhD from the University La Sapienza of Rome and completed his research training at the University Hospital in the Netherlands and the USA. He also obtained a master’s degree in intensive cardiac care from the Univerisity La Cattolica and in healthcare management from the Luiss Buisiness School of Rome. He is the vice president of the Italian Association of Hospital Cardiologists (ANMCO) and a member of the research section of the ESC-ACC (Association of Acute Cardiovascular Care).

## Supplementary Material

oead076_Supplementary_Data

## Data Availability

The data that support the findings of this study are available from the corresponding author upon reasonable request.
