# Supplementary material for: Safety of cangrelor and transition to oral P2Y12 inhibitors in patients undergoing percutaneous coronary intervention: the ARCANGELO study
Source: Eur Heart J Open. 2023 Aug 28;3(4):oead076. doi: 10.1093/ehjopen/oead076 (PMC10462400; doi:10.1093/ehjopen/oead076)
Supplement: oead076_Supplementary_Data [file oead076_supplementary_data.docx]

# Supplemental material

[Supplemental material 1](#_Toc139038987)

[**The ARCANGELO Study Group** 1](#_Toc139038988)

[**Supplementary tables** 3](#_Toc139038989)

## **The ARCANGELO Study Group**

Paolo Calabrò (U.O.C. Cardiologia Clinica con UTIC. A.O.R.N. Sant'Anna e San Sebastiano, Caserta); Fabio Chirillo (U.O.C. Cardiologia, Ospedale San Bassiano, Bassano del Grappa (VI); Cristina Rolfo (S.C. Cardiologia Ospedale degli Infermi di Rivoli ASLTO); Alberto Menozzi (S.C. Cardiologia, Ospedale S. Andrea, La Spezia, ASL5 Liguria); Piera Capranzano (U.O. Cardiologia A.O.U. Policlinico G. Rodolico, Catania); Maurizio Menichelli (Cardiologia Ospedale Fabrizio Spaziani, Frosinone); Elisa Nicolini (U.O. Cardiologia Interventistica, strutturale e pediatrica, Ospedali Riuniti Ancona); Ciro Mauro (Cardiologia UTIC con emodinamica A.O.R.N. Cardarelli Napoli); Carlo Trani (U.O.C. Interventistica Cardiologica e diagnostica invasiva, Fondazione Policlinico Universitario A. Gemelli, IRCCS, Rome); Francesco Versaci (Department of Cardiology, Santa Maria Goretti Hospital, Latina); Fabrizio Tomai (U.O.C. di Cardiologia Aurelia Hospital); Giuseppe Musumeci (S.C. Cardiologia, A.O. Ordine Mauriziano, Torino); Leonardo De Luca (Cardiologia UTIC Ospedale San Camillo Forlanini, Rome); Carlo Di Mario (Interventistica Cardiologica Strutturale A.O.U. Careggi, Firenze); Martino Pepe (Cardiologia Universitaria A.O.U. Consorziale Policlinico); Sergio Berti (Fondazione C.N.R. Reg. Toscana G. Monasterio, Pisa); Carlo Cernetti (Cardiologia Ospedale Ca' Foncello, Treviso); Plinio Cirillo (Dipartimento di Scienze Biomediche Avanzate, Cardiologia, A.O.U.P. “Federico II”, Napoli); Diego Maffeo (Cardiologia Emodinamica Fondazione Poliambulanza, Brescia); Talanas (U.O.C. Cardiologia Clinica ed Interventistica Ospedale SS Annunziata, Sassari); Marco Ferlini (U.O.C. Cardiologia Fondazione IRCCS Policlinico San Matteo, Pavia); Marco Contarini (U.O.C. di Cardiologia con UTIC ed Emodinamica Ospedale Umberto I di Siracusa Azienda Sanitaria Provinciale di Siracusa); Valerio Lanzilotti (U.O.C. Cardiologia Ospedale Maggiore, Bologna); Marino Scherillo (U.O.C. Cardiologia interventistica e UTIC Azienda Ospedaliera San Pio, Benevento); Giuseppe Tarantini (U.O.S.D. Emodinamica e Cardiologia Interventistica Azienda Ospedale Università Padova); Simone Muraglia (U.O. Cardiologia, Ospedale S. Chiara, Trento); Roberta Rossini (Cardiologia ASO Santa Croce e Carle, Cuneo); Leonardo Bolognese (Cardiologia Ospedale San Donato, Arezzo).

## **Supplementary tables**

Supplementary Table 1 - Reason for choosing cangrelor

| **Reason for choosing cangrelor** | |
| --- | --- |
| Due to urgency of PCI | 888 ( 89.2%) |
| Patient with nausea/vomit | 42 ( 4.2%) |
| Difficulties in swallowing | 25 ( 2.5%) |
| Cardiogenic shock | 20 ( 2.0%) |
| Intubation and ventilation | 16 ( 1.6%) |
| Not Pre-Treated With P2Y12 | 16 ( 1.6%) |
| Possible cardiac surgery | 12 ( 1.2%) |
| High Thrombus Burden | 6 ( 0.6%) |
| Strong sedation | 4 ( 0.4%) |
| Routine Use | 2 ( 0.2%) |
| Aspirin Intolerance | 1 ( 0.1%) |
| Complex PCI (Left Main Artery Involved) | 1 ( 0.1%) |

**Note. Multiple answers were allowed.**

Supplementary Table 2 - Location of bleeding

| **System organ class** | **Preferred term** | **BARC type 1-2 bleedings (N=50)** | **BARC type 3-5 bleedings (N=5)** | **Total bleedings (N=55)** |
| --- | --- | --- | --- | --- |
| **Number of bleeding events** | **Any** | 50 (100.0%) | 5 (100.0%) | 55 (100.0%) |
| **Gastrointestinal disorders** | **Any** | 5 (10.0%) | 0 (0.0%) | 5 (9.1%) |
|  | **Gingival bleeding** | 2 (4.0%) | 0 (0.0%) | 2 (3.6%) |
|  | **Haematochezia** | 1 (2.0%) | 0 (0.0%) | 1 (1.8% |
|  | **Haemorrhoidal haemorrhage** | 1 (2.0%) | 0 (0.0%) | 1 (1.8%) |
|  | **Lower gastrointestinal haemorrhage** | 1 (2.0%) | 0 (0.0%) | 1 (1.8%) |
| **General disorders and administration site conditions** | **Any** | 6 (12.0%) | 3 (60.0%) | 9 (16.4%) |
|  | **Vessel puncture site haematoma** | 3 (6.0%) | 1 (20.0%) | 4 (7.3%) |
|  | **Catheter site haematoma** | 1 (2.0%) | 2 (40.0%) | 3 (5.5%) |
|  | **Catheter site haemorrhage** | 1 (2.0%) | 0 (0.0%) | 1 (1.8%) |
|  | **Vessel puncture site haemorrhage** | 1 (2.0%) | 0 (0.0%) | 1 (1.8%) |
| **Investigations** | **Any** | 0 (0.0%) | 1 (20.0%) | 1 (1.8%) |
|  | **Haemoglobin decreased** | 0 (0.0%) | 1 (20.0%) | 1 (1.8%) |
| **Renal and urinary disorders** | **Any** | 6 (12.0%) | 1 (20.0%) | 7 (12.7%) |
|  | **Haematuria** | 5 (10.0%) | 1 (20.0%) | 6 (10.9%) |
|  | **Urethral haemorrhage** | 1 (2.0%) | 0 (0.0%) | 1 (1.8%) |
| **Respiratory, thoracic and mediastinal disorders** | **Any** | 6 (12.0%) | 0 (0.0%) | 6 (10.9%) |
|  | **Epistaxis** | 6 (12.0%) | 0 (0.0%) | 6 (10.9%) |
| **Skin and subcutaneous tissue disorders** | **Any** | 17 (34.0%) | 0 (0.0%) | 17 (30.9%) |
|  | **Ecchymosis** | 12 (24.0%) | 0 (0.0%) | 12 (21.8%) |
|  | **Skin haemorrhage** | 3 (6.0%) | 0 (0.0%) | 3 (5.5%) |
|  | **Haemorrhage subcutaneous** | 2 (4.0%) | 0 (0.0%) | 2 (3.6%) |
| **Vascular disorders** | **Any**  **Arterial haemorrhage**  **Haematoma** | 10 (20.0%)  6 (12.0%)  2 (4.0%) | 0 (0.0%)  0 (0.0%)  0 (0.0%) | 10 (18.2%)  6 (10.9%)  2 (3.6%) |
|  | **Haemorrhage** | 1 (2.0%) | 0 (0.0%) | 1 (1.8%) |
|  | **Venous haemorrhage** | 1 (2.0%) | 0 (0.0%) | 1 (1.8%) |

Supplementary Table 3 – Incidence of haemorrhages (% of patients) by oral platelet P2Y12 receptor agonist

|  | | **Patients with clopidogrel**  **(N=138)** | **Patients with prasugrel**  **(N=127)** | **Patients with ticagrelor**  **(N=730)** |
| --- | --- | --- | --- | --- |
| *Patients experiencing at least one haemorrhage* | | *3 (2.2%)* | *10 (7.9%)* | *39 (5.3%)* |
| Number of any haemorrhages per patient | 0 | 135 (97.8%) | 117 (92.1%) | 691 (94.7%) |
|  | 1 | 2 (2.2%) | 10 (7.9%) | 36 (4.9%) |
|  | 2 | 0 | 0 | 3 (0.4%) |

Supplementary Table 4 – Incidence of MACEs (% of patients) at 48 hours (a) and 30 days post-PCI

|  | **48 hours** | | | **30 days** | | |
| --- | --- | --- | --- | --- | --- | --- |
|  | **STEMI**  **(N= 597)** | **NSTE-ACS**  **(N= 398)** | **Eligible patients**  **(N=995)** | **STEMI**  **(N= 597)** | **NSTE-ACS**  **(N= 398)** | **Eligible patients**  **(N=995)** |
| **At least one MACE** | 6 ( 1.0%) | 4 ( 1.0%) | 10 ( 1.0%) | 10 ( 1.7%) | 4 ( 1.0%) | 14 ( 1.4%) |
| **Death (all reasons)** | 2 ( 0.3%) | 2 ( 0.5%) | 4 ( 0.4%) | 4 ( 0.7%) | 2 ( 0.5%) | 6 ( 0.6%) |
| Cardiovascular | 1 ( 0.2%) | 0- | 1 ( 0.1%) | 2 ( 0.3%) | 0- | 2 (0.2%) |
| Non-cardiovascular | 1 ( 0.2%) | 2 ( 0.5%) | 3 ( 0.3%) | 2 ( 0.3%) | 2 ( 0.5%) | 4 ( 0.4%) |
| **MI** | 3 ( 0.5%) | 2 ( 0.5%) | 5 ( 0.5%) | 5 ( 0.8%) | 2 ( 0.5%) | 7 ( 0.7%) |
| Type 1 | 0- | 0 | 0 | 1 ( 0.2%) | 0 | 1 ( 0.1%) |
| Type 2 | 1 ( 0.2%) | 0 | 1 ( 0.1%) | 2 ( 0.3%) | 0 | 2 ( 0.2%) |
| Type 3 | 0 | 0 | 0 | 0 | 0 | 0 |
| Type 4a | 2 ( 0.3%) | 2 ( 0.5%) | 4 ( 0.4%) | 2 ( 0.3%) | 2 ( 0.5%) | 4 ( 0.4%) |
| Type 4b | 0 | 0 | 0 | 0 | 0 | 0 |
| **IDR** | 0 | 0 | 0 | 0 | 0 | 0 |
| **ST** | 1 ( 0.2%) | 0 | 1 ( 0.1%) | 2 ( 0.3%) | 0 | 2 ( 0.2%) |
| Definite ST | 1 ( 0.2%) | 0 | 1 ( 0.1%) | 2 ( 0.3%) | 0 | 2 ( 0.2%) |

**Note. The same patient could have had more events of the same type and/or more events of different types. IDR: Ischemia-driven Revascularization; MACE: Major Adverse Cardiac Events; MI; Myocardial Infarction; NSTE-ACS: Non-ST-segment Elevation Acute Coronary Syndrome; ST: Stent Thrombosis; STEMI: ST-segment Elevation Myocardial Infarction.**

Supplementary Table 5 - Incidence of Treatment-Emergent Adverse Events during observation listed by System Organ Class

| **System Organ Class** | **STEMI (N= 597)** | **NSTE-ACS (N= 398)** | **Eligible patients (N=995)** |
| --- | --- | --- | --- |
| **Patients with any TEAE related to cangrelor** | 5 (0.8%) | 1 (0.3%) | 6 (0.6%) |
| Blood and lymphatic system disorders (any) | 2 (0.3%) | 0 (0.0%) | 2 (0.2%) |
| *Anaemia* | 1 (0.2%) | 0 (0.0%) | 1 (0.1%) |
| *Thrombocytopenia* | 1 (0.2%) | 0 (0.0%) | 1 (0.1%) |
| Eye disorders (*Visual impairment*) | 1 (0.2%) | 0 (0.0%) | 1 (0.1%) |
| Gastrointestinal disorders (*Gingival bleeding)* | 0 (0.0%) | 1 (0.3%) | 1 (0.1%) |
| General disorders and administration site conditions (*Catheter site hemorrhage*) | 0 (0.0%) | 1 (0.3%) | 1 (0.1%) |
| Investigations (*Haemoglobin decreased*) | 1 (0.2%) | 0 (0.0%) | 1 (0.1%) |
| Skin and subcutaneous tissue disorders (*Ecchymosis*) | 1 (0.2%) | 0 (0.0%) | 1 (0.1%) |
| Vascular disorders (*Haematoma)* | 1 (0.2%) | 0 (0.0%) | 1 (0.1%) |

**NSTE-ACS: Non-ST-segment Elevation Acute Coronary Syndrome; STEMI: ST-segment Elevation Myocardial Infarction.**
